# Chronic Ectopic Pregnancy: A Case Report

**DOI:** 10.7759/cureus.91468

**Published:** 2025-09-02

**Authors:** Sherin M Hassaballa, Kareem Aboualfa, Nayantara Bijral

**Affiliations:** 1 Obstetrics and Gynaecology, East Kent Hospitals NHS Trust, Ashford, GBR; 2 Pathology and Laboratory Medicine, East Kent Hospitals NHS Trust, Ashford, GBR

**Keywords:** chronic ectopic pregnancy, clinical diagnosis, disparity in definition, histopathological diagnosis, surgical findings

## Abstract

Chronic ectopic pregnancy (CEP) is an under-recognised variant of ectopic pregnancy, characterised by low or absent serum beta human chorionic gonadotropin (β-hCG), a complex adnexal mass, and non-specific clinical symptoms and signs. We present a case of a woman in her 30s with progressive lower abdominal pain, an equivocal pregnancy test, and spontaneous vaginal bleeding. Transvaginal ultrasound demonstrated an inhomogeneous, avascular right adnexal mass on ultrasound, with associated haemoperitoneum. A diagnostic laparoscopy revealed a haemorrhagic ampullary mass and 300 mL of intraperitoneal blood. The right fallopian tube was excised, and histopathological examination confirmed CEP. This case highlights the complexity of obtaining a diagnosis of CEP, particularly when symptoms and investigations may mimic other pelvic pathologies. Laparoscopic salpingectomy remains the mainstay of treatment as it eliminates the risk of any persistent trophoblastic tissue. CEP should remain a differential diagnosis in any patient of reproductive age with pelvic pain or abnormal bleeding, irrespective of β-hCG levels, to avoid premature discharge and reduce delays in definitive management.

## Introduction

An ectopic pregnancy is any developing pregnancy implanted outside of the endometrial cavity of the uterus, most commonly in the ampullary region of the fallopian tube. In the UK, the incidence is approximately 11/1000 pregnancies, with an estimated 11000 diagnosed each year [1]. Chronic ectopic pregnancy (CEP), one of its variants, remains a poorly understood and under-recognised entity.

CEP is characterised by an adnexal mass causing progressive disintegration of the tubal wall with slow, repeated episodes of minor rupture. Clinically, symptoms such as pelvic pain and irregular vaginal bleeding are common, but 18% of patients are asymptomatic [2]. Serum beta human chorionic gonadotropin (β-hCG) is often low or undetectable due to the lack of functioning trophoblastic tissue [3]. Histologically, it is defined by the presence of multiple blood clots interspersed with degenerated chorionic villi, often encased within dense fibrous adhesions [4]. Chronic inflammation is also a hallmark of CEP, with leukocyte infiltration, abscess formation, and elevated inflammatory markers all being reported in the literature [3,5].

Due to the disparity in definitions, the true incidence of CEP is difficult to establish. Estimates widely vary, with reported incidence ranging from 6% to 44% of all ectopic pregnancies [3,4,6,7]. However, there is no reliable national incidence data, and CEP is notably absent from the Royal College of Obstetrics and Gynaecology Green-Top Guideline on the diagnosis and management of ectopic pregnancy [1].

This case aims to highlight the diagnostic complexity of CEP and reinforce its inclusion in the differential diagnoses of pelvic pain, vaginal bleeding or adnexal mass in individuals of reproductive age, even in the context of a negative or equivocal serum or urine β-hCG. There were 12 maternal deaths from ectopic pregnancy in 2021 and 2022, and assessors felt that improvements to care would have made a difference to 75% of these women [8]. Clinicians of all levels should be supported to undertake a holistic assessment of these patients and seek early involvement from senior colleagues.

## Case presentation

A woman in her 30s, gravida 5 para 2+2, was brought to the emergency department by ambulance with waves of progressively worsening lower abdominal pain. She had eight weeks of amenorrhoea and a faintly positive home pregnancy test. This was followed by a single episode of heavy vaginal bleeding, which resolved spontaneously, and was succeeded by persistent brown discharge. Believing she had experienced a miscarriage, she did not seek medical attention at the time.

She had two uncomplicated term vaginal deliveries and two surgical terminations for pregnancy. She was a smoker with a body mass index of 26 kg/m^2^. Her medical history was unremarkable, apart from anxiety disorders.

She was haemodynamically stable. She had suprapubic and bilateral iliac fossa tenderness, without guarding or rebound.

Her Inflammatory markers (Table 1) showed increased white blood cell count (WCC) with normal C-reactive protein (CRP). Serum β-hCG was low (Table 1).

**Table 1:** Patient's blood test results. Inflammatory markers showed increased white blood cell count (in keeping with an inflammatory response) with normal CRP. Serum beta human chorionic gonadotropin (β-hCG) was weakly positive (in keeping with degeneration or absence of active trophoblastic tissue).

Type of blood test	Patient	Reference range
White blood cell count (WCC)	18.1 x 10^9/L	4.5-11.0 x 10^9/L
C-reactive protein (CRP)	1 mg/L	0-5 mg/L
Serum β-hCG	10 IU/L	Less than 5 IU/mL

**Figure 1:**
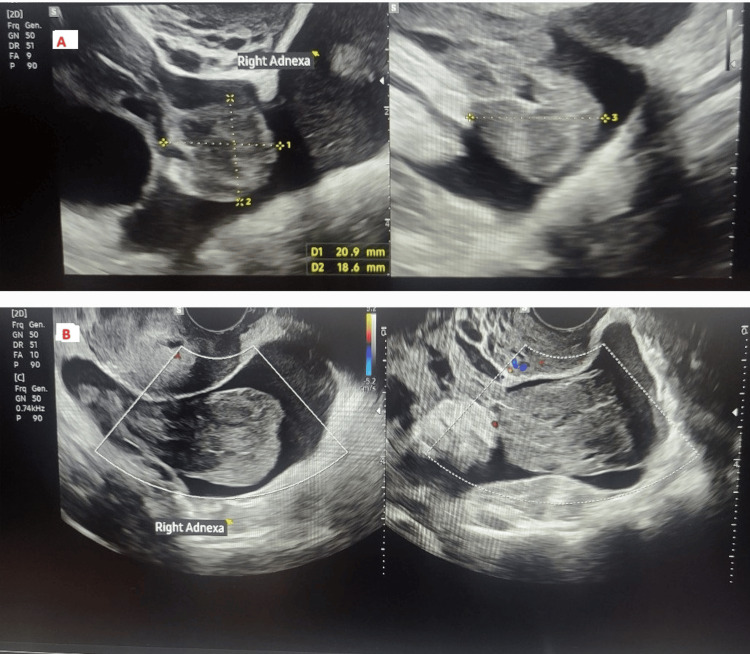
Transvaginal ultrasound demonstrated an inhomogeneous ectopic mass (A) in the sagittal and (B) coronal plane. Inhomogeneous, hyperechoic mass measuring 20.9 x 18.6 x 19.1 mm adjacent to the right ovary, lacking internal vascularity on colour Doppler.

Transvaginal ultrasound demonstrated an inhomogeneous, hyperechoic mass measuring 20.9 x 18.6 x 19.1 mm adjacent to the right ovary, lacking internal vascularity on colour Doppler (Figure 1). Surrounding this, mixed echogenic free fluid was observed. Within the fluid, a homogenous mass measuring 60.5 x 40.2 x 28.3 mm was visualised, likely representing a blood clot. The uterus was anteverted with normal size, shape, and echo texture. The endometrial thickness was 9.8 mm with a trilaminar pattern. The uterine cavity was empty. Anechoic free fluid in the pouch of Douglas measured 47.2 mm in depth. The absence of an intrauterine pregnancy and an equivocal human chorionic gonadotropin (hCG) level did indeed raise the possibility of a failing pregnancy, miscarriage, or pregnancy of unknown location. However, the presence of a complex adnexal mass and free fluid kept the suspicion of an ectopic process in play.

**Figure 2:**
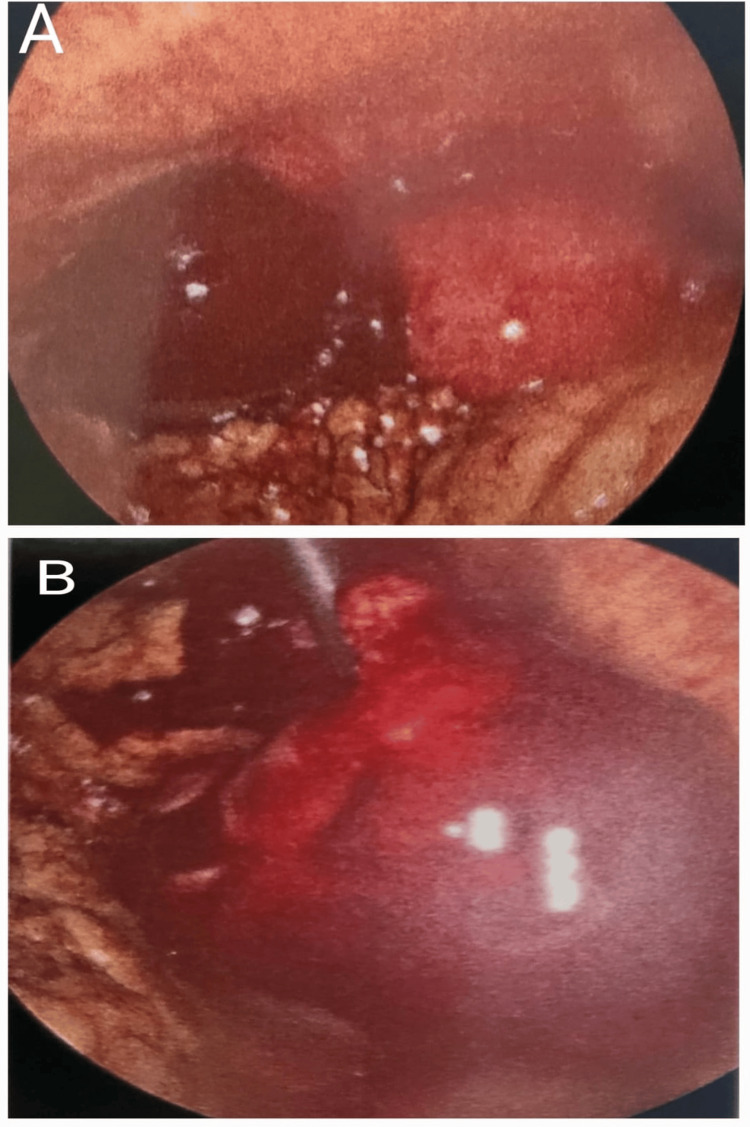
Laparoscopic findings. (A) Left tubal ectopic or haematosalpinx with 300 ml of blood in the peritoneal cavity. (B) Closer view of the left tubal haematosalpinx.

A diagnostic laparoscopy was performed, revealing a haemorrhagic mass in the ampullary portion of the left fallopian tube, surrounded by approximately 300 ml of haemoperitoneum (Figure 2).

**Figure 3:**
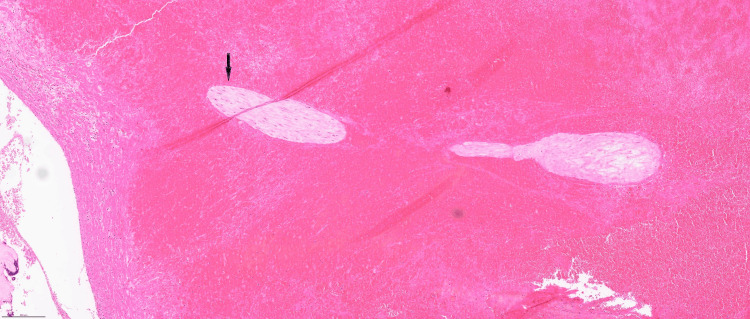
Partly autolysed chorionic villi and organised blood clot within the fallopian tube lumen. Haematoxylin and eosin stain (original magnification, x5).

**Figure 4:**
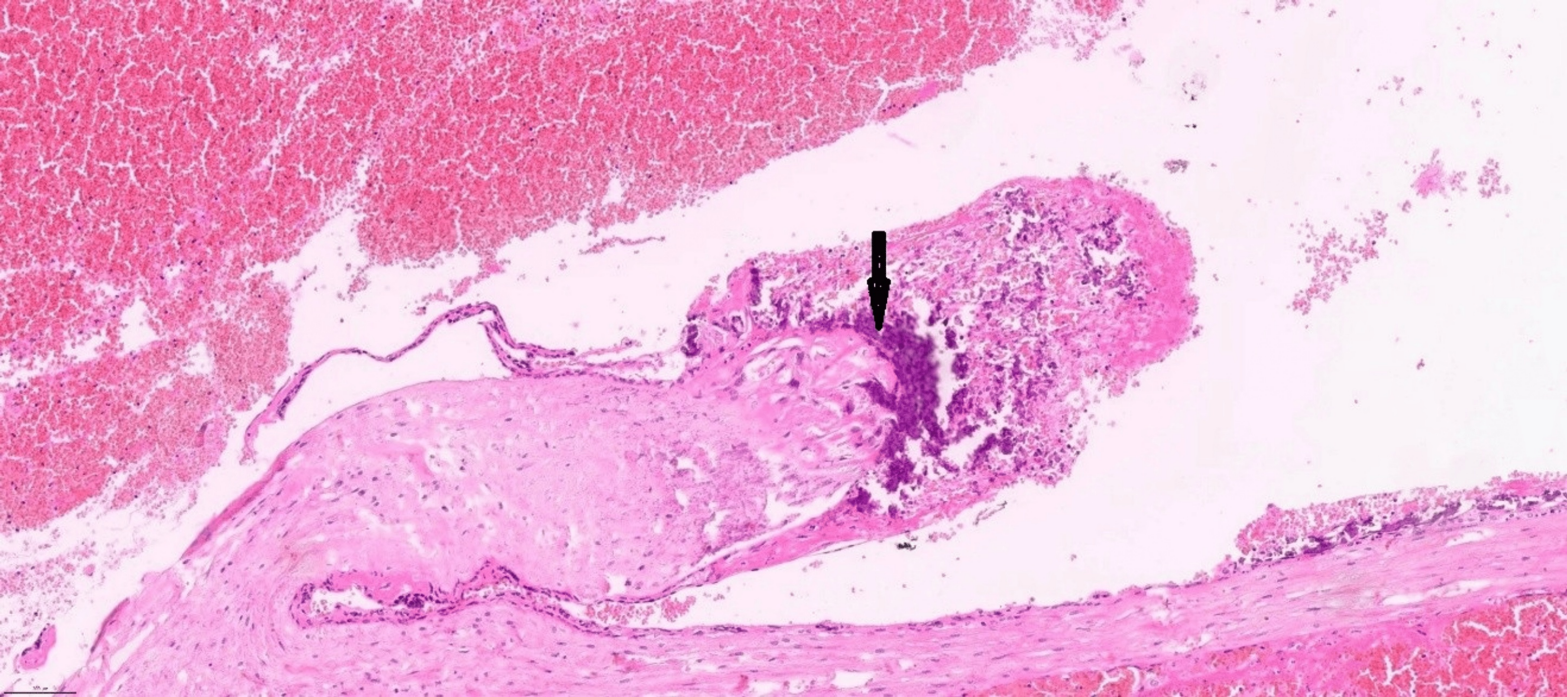
Partly autolysed chorionic villi with calcification (arrow). Haematoxylin and eosin stain (original magnification, x10).

Histological examination (Figure 3) showed partly autolysed chorionic villi and organised blood clot within the fallopian tube lumen, and partly autolysed chorionic villi with calcification (Figure 4).

She had an unremarkable recovery following surgery, with an undetectable serum β-hCG on follow-up.

## Discussion

CEP is characterised by low or absent serum β-hCG and the presence of an adnexal mass composed of degenerated trophoblastic tissue, fibrosis, necrosis, and thrombi, which is the result of repeated minor tubal ruptures leading to a haematocele. The chronic inflammatory response that ensues often results in adhesions involving adjacent pelvic and abdominal structures.

Although our patient had only one traditional risk factor for ectopic pregnancy (cigarette smoking), this case reaffirms that CEP can occur even in the absence of multiple high-risk features, in concordance with a previous multivariable analysis [7,9]. CEP presents with non-specific sub-acute symptoms, namely, pelvic pain, abnormal vaginal bleeding, and amenorrhoea. In a recent systematic review of 399 patients with CEP, 78% reported abdominal pain and 55% experienced abdominal bleeding, whilst 18% of patients were asymptomatic [2]. Due to this, there are multiple reported cases of CEP being masqueraded by other similarly presenting pathologies, including ovarian cysts and tumours, broad ligament fibroids, pelvic malignancy, and tubo-ovarian abscesses [10-14]. The intermittent nature of our patient’s symptoms and the initial resolution of bleeding demonstrate how CEP may be misdiagnosed as miscarriage and thus contribute to a delay in diagnosis.

Low or negative hCG does not exclude ectopic pregnancy. Approximately 1% of ectopic pregnancies will have a negative urine pregnancy test, and CEP has been associated with negative serum β-hCG in 32% of cases [2,15]. This is due to degeneration or absence of active trophoblastic tissue and a low chorionic villous burden. Serum β-hCG was 10 IU/L in our patient, which is too low for a viable pregnancy, but concerning for an ectopic process when interpreted with our clinical and ultrasound findings. She also had a raised WCC, which was in keeping with an inflammatory response that is seen in ectopic pregnancies generally, due to local tissue necrosis and haemorrhage. Her CRP, however, was normal. The adnexal mass was inhomogeneous and avascular on colour Doppler, findings consistent with those reported by Bedi et al. and Turan et al., who observed similar echotexture and absent Doppler flow in all cases [4,6]. These features reflect the lack of trophoblastic activity. While MRI has been proposed as a helpful adjunct, particularly in visualising haemoperitoneum and enhancing tubal walls, its utility is limited by availability, cost, and lack of necessity when surgical intervention is the mainstay of treatment of CEP [16].

Laparoscopic salpingectomy is curative, as it eliminates the risk of persistent trophoblast, and was performed successfully in our case [17]. However, CEP has previously presented with technical challenges perioperatively owing to dense adhesions involving the bowel, omentum, bladder, or contralateral adnexa [10]. Careful preoperative planning, involving general surgical input where needed, may be necessary to manage complex cases safely. While methotrexate is an established treatment option for low-risk ectopic pregnancies, its role in CEP is limited. Methotrexate is a folinic acid antagonist that blocks DNA and, to some extent, RNA synthesis and cell division. As a result, tissues with a rapid cellular turnover, such as trophoblasts, are most susceptible to its action. The absence of viable trophoblastic tissue means there is a limited biological target for methotrexate to act upon. Additionally, monitoring for resolution will be difficult, as β-hCG is often low or negative in CEP.

The histological diagnosis of a fallopian tube ectopic pregnancy requires the identification of chorionic villi within the fallopian tube tissue, as this confirms implantation outside the uterus, distinguishing ectopic pregnancy from other causes of tubal damage [18]. Despite no definitive histological criteria for chronic ectopic pregnancies, certain features can suggest chronicity or prolonged duration. In our case, the chorionic villi appeared somewhat autolysed with loss of the surrounding trophoblastic layer, and occasional calcification of villi was observed. Other studies described multiple blood clots embedded within degenerated chorionic villi and surrounded by dense adhesions as features of CEP [2].

## Conclusions

CEP is a distinct subtype of ectopic pregnancy characterised by low or absent trophoblast activity. Its clinical, biochemical, and imaging findings are often non-specific, and crucially, a negative urine or serum β-hCG does not rule it out. A high level of suspicion is required when evaluating patients of reproductive age with vague pelvic symptoms. We presented our case of CEP as an unusual presentation of the ectopic pregnancy to increase clinicians' awareness of this subtype. Laparoscopic salpingectomy is the mainstay of management, and diagnosis is confirmed with histopathological analysis. Gynaecologists should adopt a cautious and structured approach to the diagnosis and management of CEP, with the aim of preventing premature discharge, minimising misdiagnosis, and ultimately improving patient outcomes.
